# An Electrically Tunable Zoom System Using Liquid Lenses

**DOI:** 10.3390/s16010045

**Published:** 2015-12-31

**Authors:** Heng Li, Xuemin Cheng, Qun Hao

**Affiliations:** 1 School of Optoelectronics, Beijing Institute of Technology, Beijing 100081, China; liheng@bit.edu.cn; 2 Graduate School at Shenzhen, Department of Precision Instrument, Tsinghua University, Shenzhen 518055, China; cheng-xm@mail.tsinghua.edu.cn

**Keywords:** optical imaging, liquid lens, stabilized zoom system, Gaussian bracket

## Abstract

A four-group stabilized zoom system using two liquid lenses and two fixed lens groups is proposed. We describe the design principle, realization, and the testing of a 5.06:1 zoom system. The realized effective focal length (EFL) range is 6.93 mm to 35.06 mm, and the field of view (FOV) range is 8° to 40°. The system can zoom fast when liquid lens 1’s (L_1_’s) optical power take the value from 0.0087 mm^−1^ to 0.0192 mm^−1^ and liquid lens 2’s (L_2_’s) optical power take the value from 0.0185 mm^−1^ to −0.01 mm^−1^. Response time of the realized zoom system was less than 2.5 ms, and the settling time was less than 15 ms.The analysis of elements’ parameters and the measurement of lens performance not only verify the design principle further, but also show the zooming process by the use of two liquid lenses. The system is useful for motion carriers e.g., robot, ground vehicle, and unmanned aerial vehicles considering that it is fast, reliable, and miniature.

## 1. Introduction

The optical zoom system used for imaging or projection is widely applied to many fields, such as surveillance, medicine, aviation, and aerospace [1,2,3,4,5]. Optical zoom systems used in imaging must satisfy two basic conditions: adjustable focal length and a fixed image plane. Traditional zoom systems usually comprise several lens groups and the separation between adjacent groups is allowed to vary. Focal length of the zoom system is varied continuously by the displacements of lens groups, the individual lens group must move along the precise track, and the action of multiple lens groups must ensure synchronization [6]. As a result, a complex mechanical camera system is required in the process of zooming. As the conventional zoom system is applied to high-speed moving vehicles, the reliability of the conventional zoom systems will be reduced due to vibration, and the response time is hard to meet the requirement of high-speed as well.

A novel optical zoom system based on focal length variable optical components has been presented. Focal length of such optical components can be adjusted by varying its surface shape or refractive index [7,8]. Different from traditional optical zoom systems, this new type of optical zoom system can operate by adjusting the focal length variable optical components without involving any motorized movements. This novel optical zoom system is more attractive because of the low complexity, high reliability, and the ability to be easily miniaturized, which make it suitable for being utilized in robots or other moving carriers. Many researchers designed zoom systems based on such focal length variable optical components, which reduced the dependence on motorized components, therefore effectively decreasing the complexity of the system [9,10,11,12,13,14,15,16,17]. However, there still remain difficulties in the realization of stabilized zoom systems using optical power as a zoom variable. For example, it would be ideal if the focal length variable elements would compensate for the image shift while taking over the variations of the focal length of the zoom system. This is one reason why most of the existing designs still have a small amount of motorized components. Requirements in large zoom ratio, high zoom precision, high imaging quality, and excellent electrical performance are also giving lens design greater challenges.

In this paper, we propose to realize a four-group stabilized zoom system based on two liquid lenses. Firstly, we analyze the solution of image displacement compensation, and discuss the design method based on the theory of Gaussian brackets. Stable image plane provides help for improving the imaging quality. The method has been presented in our previous work [18]. It solved the problem of increasing zoom ratio with a mathematical method. Optimization of parameters was realized by mathematically solving extreme value and maximum gradient of the function. Application of the method has been extended in this paper. In view of the specific application, we designed and optimized a realizable zoom system, and the zoom ratio is designed to be 5:1. Secondly, we realized the zoom device and set up an experiment platform to test the performance of the zoom system. We test zoom ratio, zoom precision, and imaging quality. The results show that the realized system has good electrical performance and high imaging quality. Zoom ratio and precision of the system have reached the design target.

## 2. Theory Analysis and Design

### 2.1. Imaging System Design

**Figure 1:**
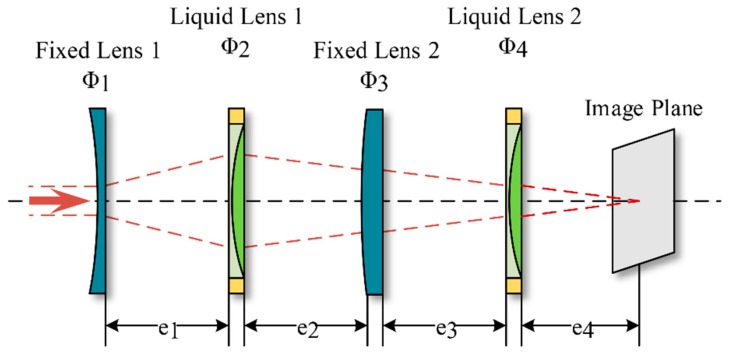
Structure of the zoom system.

We presented a design method that is applicable to four-group stabilized zoom system in the previous work [18]. The optical layout of the four-group stabilized zoom system is shown in Figure 1, where Φ_1_ and Φ_3_ are the optical power of the fixed groups, Φ_2_ and Φ_4_ are optical power of the stabilized focal-length-variable groups. The terms e_1_, e_2_, e_3_ are the separations between the consecutive principle planes in each group, e_4_ is the separation between the last group and the image plane, m_i_ is magnification of each group.

In previous work, we analyzed this four-group stabilized zoom systems using the optical power of the optical element as the independent variables in the zoom equation. Simplified differential function is used to describe the relationship between the focal length variation of varifocal elements and the focal length variation of zoom system. The differentiable condition optimization can be used to get the analytic solution. In the zooming process, the function of the optical power of the focal length variable groups is monotonic, and the function of magnification has a breakpoint. The optical layout can be miniaturized as magnification has a larger derivative near the breakpoint. If the sign of m_3_ is changeable during zooming, a larger derivative of m_3_ can be found near the breakpoint. Then we optimize the Gaussian parameters of the fixed lens groups, make $\frac{e_{4}}{(1-e_{1}\phi_{1})\cdot {{\;}^{2}B}_{4}}$ and $\frac{1-e_{1}\phi_{1}}{e_{4}\cdot {{\;}^{2}B}_{4}}$ (B42=[−e2,ϕ3,−e3]=e2⋅e3⋅ϕ3−e2−e3 is Gaussian brackets parameter) converge to a minimum to reach the requirement of zoom ratio.

In this paper, the optical design of a 5:1 stabilized zoom system is investigated. The zoom range is designed to be 7 mm to 35 mm. Design parameters are determined based on the requirement of a kind of ground vehicle being deployed on a bumpy road. The values of the Gaussian parameters are listed in Table 1, $\left|\frac{e_{4}}{(1-e_{1}\phi_{1})\cdot {{\;}^{2}B}_{4}}\right|=0.12$, and $\left|\frac{1-e_{1}\phi_{1}}{e_{4}\cdot {{\;}^{2}B}_{4}}\right|=0.001$.

**Figure 2:**
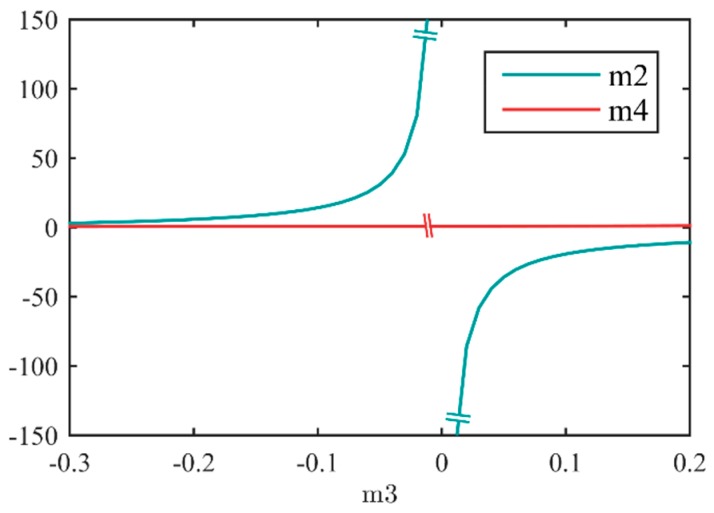
Relationship between m_2_, m_3_, and m_4_. A larger derivative of m_3_ can be found near the breakpoint since the sign of m_3_ is changeable.

The relationship between m_2_, m_3_ and m_4_ is shown in Figure 2, a larger derivative of m_3_ can be found near the breakpoint since the sign of m_3_ is changeable.

**Table 1:** Gaussian parameters of the zoom system.

	1/Φ (mm)	Φ_2_ (mm^−1^)	Φ_4_ (mm^−1^)	m_2_	m_3_	m_4_
Wide	7.27	0.008	0.019	1.28	−0.28	0.56
Middle	26.14	0.019	0	2.31	0.11	1.03
Tele	36.34	0.02	−0.01	2.53	0.19	1.24

We use two liquid lenses (L_1_ and L_2_) as the focal length variable groups (Φ_2_, Φ_4_), the variation of Φ_2_ and Φ_4_ can be obtained as follows:

(1) $$\Phi_{2}=\frac{e_{4}\Phi -[\phi_{1},-(e_{1}+e_{2}),\phi_{3},-e_{3}]}{(1-e_{1}\phi_{1})\cdot {{\;}^{2}B}_{4}}$$

(2) $$\Phi_{4}=\frac{1-e_{1}\phi_{1}}{e_{4}\Phi \cdot {{\;}^{2}B}_{4}}+\frac{{{\;}^{2}B}_{4}-e_{4}[-e_{2},\phi_{3}]}{e_{4}\cdot {{\;}^{2}B}_{4}}$$

(3) $$\Delta \Phi_{2}=\frac{e_{4}}{(1-e_{1}\phi_{1})\cdot {{\;}^{2}B}_{4}}\Delta \Phi$$

(4) $$\Delta \Phi_{4}=\frac{1-e_{1}\phi_{1}}{e_{4}\cdot {{\;}^{2}B}_{4}}\Delta \left(\frac{1}{\Phi }\right)$$

where $\Delta \Phi =\Phi_{s}-\Phi_{l}$ is the variation of the system optical power, $\Delta \left(\frac{1}{\Phi }\right)=\frac{1}{\Phi_{s}}-\frac{1}{\Phi_{l}}$, Φ_s_ and Φ_l_ represent the system optical power at the wide-angle end and the telephoto end respectively, ∆Φ_2_ and ∆Φ_4_ are the variation needed to achieve a given zoom range $\frac{1}{\Phi_{s}}$ to $\frac{1}{\Phi_{l}}$.

**Figure 3:**
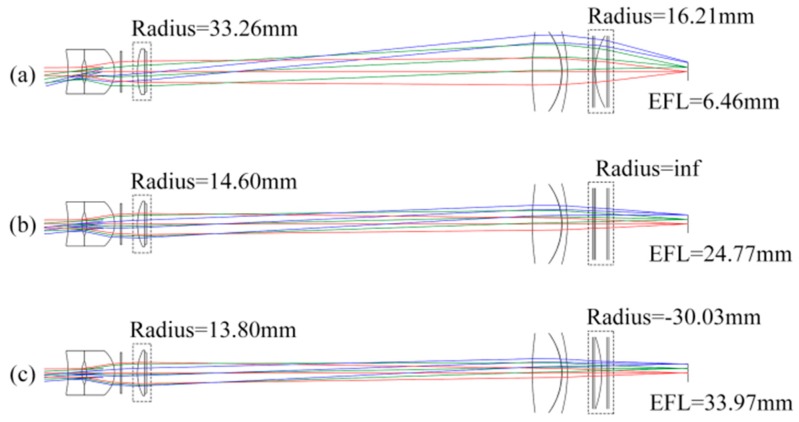
Initial lens structure of the optical system.

The general parameters of the zoom system are shown in Table 2. The initial lens structure of the optical system using these Gaussian parameters is simulated and optimized with the commercial optical design software named CODE V (Optical Research Associates, Tucson, AZ, USA), the result is shown in Figure 3, as the wide-angle end, the middle phase, and the telephoto end are shown in (a), (b), and (c), respectively.

**Table 2:** General parameters of the zoom system.

Surface	#	Curvature Radius	Thickness	Glass
Object		Infinity	Infinity	
	1	Infinity	−9.7160	
Fixed Lens1	2	−27.4979	3.7885	HLAF4_CDGM
	3	7.5970	1.4003	
	4	−9.0978	7.8405	ZK9_CHINA
	5	−8.1539	1.5000	
	6	Infinity	0.1000	
Liquid Lens1	7	Infinity	0.5000	BK7_SCHOTT
	8	Infinity	zoom	
	9	zoom	zoom	“OL1024”
	10	Infinity	0.5000	BK7_SCHOTT
	11	Infinity	1.1000	
	12	Infinity	25.9971	
	13	Infinity	81.4883	
Fixed Lens2	14	77.7631	8.2500	ZK9_CHINA
	15	−18.8293	0.1000	
	16	−18.5589	1.5000	ZF50_CDGM
	17	−41.6000	7.0723	
	18	Infinity	0.0500	
Liquid Lens2	19	Infinity	0.5000	BK7_SCHOTT
	20	Infinity	zoom	
	21	zoom	zoom	“OL1024”
	22	Infinity	0.5000	BK7_SCHOTT
	23	Infinity	3.4500	
	24	Infinity	0.8943	
	25	Infinity	17.9482	
Image		Infinity	0.0000	

### 2.2. Operating Range and Precision

**Figure 4:**
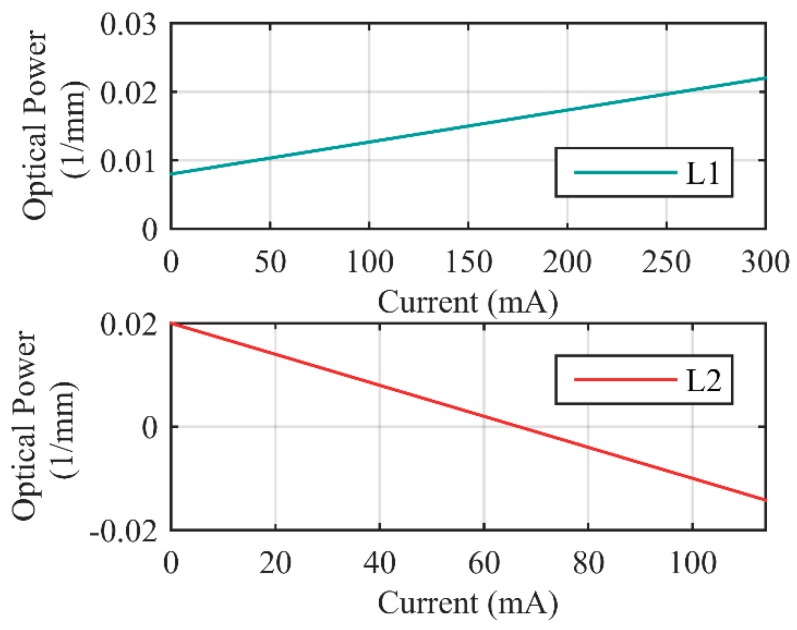
Optical power of the liquid lenses as functions of control currents.

The zoom system operates by changing the optical power of two liquid lenses (L_1_’s optical power Φ_2_, and L_2_’s optical power Φ_4_). Two focus tunable lenses (Optotune, Swizerland) are used in the experiments, a 10 mm aperture commercial lens EL-10-30 for L_1_, and a 20 mm aperture custom-design lens for L_2_. Both L_1_ and L_2_ are liquid-filled membrane lenses, the refractive index of the lenses is 1.30, and Abbe number is 100. Φ_2_ and Φ_4_ are controlled by adjusting L_1_’s control current (I_1_) and L_2_’s control current (I_2_), respectively. Range of Φ_2_ is 0.008 mm^−1^ to 0.022 mm^−1^, control precision is 4.67 × 10^−5^ mm^−1^. Range of Φ_4_ is −0.0142 mm^−1^ to 0.02 mm^−1^, the control precision is 3 × 10^−4^ mm^−1^, zero optical power is attainable. The relationships between optical power and control currents are shown in Figure 4. As shown in figure, precision of L_1_ is much higher than L_2_, thus we use L_2_ as active element, and use L_1_ as servo element to compensate the image displacement.

**Figure 5:**
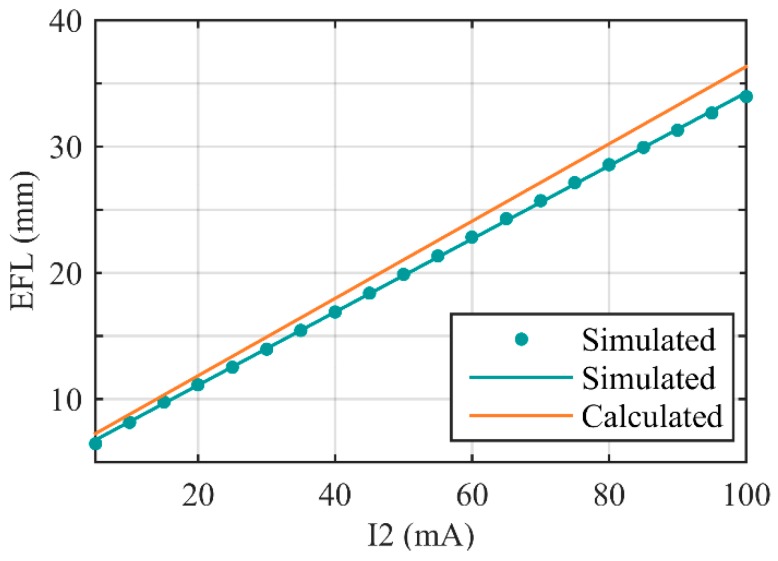
Calculated and simulated EFL as functions of I_2_. The “simulated dots” mark the EFL of sampling points that be obtained by CODE V. The “simulated line” is the fitting curve of the dots.

Our purpose is to design a zoom system with effective focal length (EFL) of 7 mm to 35 mm. In Section 2.1, we calculated the Gaussian parameters of the system, and optimized the system with CODE V. From Equation (2), we can calculate EFL of the zoom system theoretically. The calculated range of EFL is 7.27 mm to 36.34 mm, the zoom ratio is 5:1. Then we simulate the designed system, select 20 sampling points, set I_2_ from 5 mA to 100 mA, respectively. Relationships between EFL and I_2_ are shown in Figure 5. The simulated EFL range is 6.46 mm to 33.97 mm, and the zoom ratio is 5.26:1.

In this paper, zoom precision is defined as the minimum variation of EFL that can be adjusted *i.e.*, the variation of EFL when I_2_ increase or decrease 1 mA. From Equation (4), we can theoretically calculate the zoom precision to be 0.3 mm. Then we simulate the zoom precision with CODE V. We firstly select 20 sampling points when I_2_ takes different values listed in Table 3, then make I_2_ increase 1 mA, corresponding values of Φ_4_ can be obtained from Figure 4. The simulated results are shown in Table 3.

**Table 3:** Simulated zoom precision of the zoom system.

I_2_ (mA)	Zoom Precision (mm)	I_2_ (mA)	Zoom Precision (mm)
5	0.2911	55	0.2945
10	0.2731	60	0.2924
15	0.2612	65	0.2898
20	0.2762	70	0.2867
25	0.2862	75	0.2832
30	0.292	80	0.2792
35	0.295	85	0.2749
40	0.2964	90	0.2703
45	0.2966	95	0.2652
50	0.2959	100	0.2601
Average	0.2830	RMS	0.0122

Therefore, we designed and simulated a zoom system with 5:1 zoom ratio. The calculated EFL range is 7.27 mm to 36.34 mm while the simulated range is 6.46 mm to 33.97 mm. The calculated zoom precision is 0.3 mm while the average simulated value is 0.2830, and root-mean-square (RMS) of the simulated zoom precision is 0.0122 mm.

## 3. Experimental and Analysis

### 3.1. Experimental Setup

**Figure 6:**
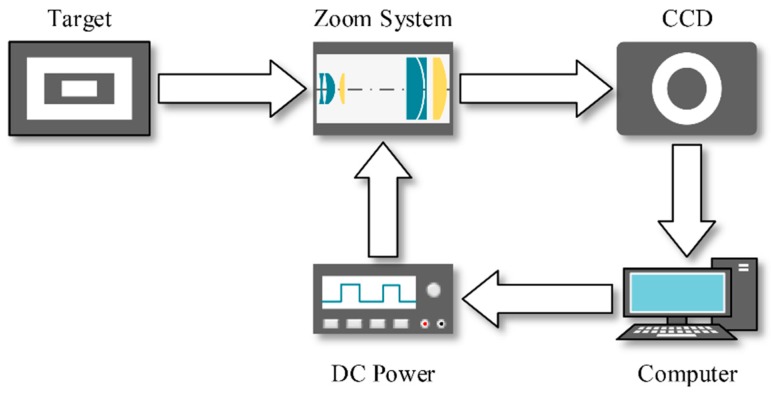
Structure of the experimental platform.

In order to test the performance of the zoom system, we set up an experimental platform shown in Figure 6. Test targets were backlit with white light and two forms of targets were used in measurement. The size of the target was 240 mm × 170 mm. The zoom system was placed between the test target and the CCD camera, and the object distance was set to be 265 mm. The CCD was 1/4 inch, pixel size of which was 7.4 μm. In fact, the zoom system and the CCD camera were fixed connected through a standard C lens mount. CCD camera captured the images and then sent them to the computer. We obtained the information on imaging quality and magnification through image processing, and then sent new control parameters to the zoom system. A dc power supply was used to complete the D/A conversion.

There were two major parameters we needed to measure: EFL and modulation transfer function (MTF). EFL was used to evaluate the zoom ratio and zoom precision, and MTF was used to evaluate the imaging quality. During measurement, we selected 20 sampling points as we had done in simulation when adjusted I_2_ from 5 mA to 100 mA. The forms of targets and the methods of measuring EFL and MTF would be discussed in the following sections.

### 3.2. Initial Structure

In their initial state, I_1_ and I_2_ both took 0 mA. MTF was used to evaluate the quality of imaging. The method of calculating MTF of the system would be provided in Section 3.6, we currently used the calculation result. Initial parameters of the zoom system are shown in Table 4. MTF value when spatial frequency took 20 lp/mm was used to evaluate the image quality.

**Table 4:** Initial parameters of the zoom system.

I_2_ (mA)	EFL (mm)	FOV (Degree)	MTF Value at 20l p/mm
0	6.56	41.7	0.01
5	6.93	39.7	0.28

Image quality was poor because L_1_ had not compensated for L_2_ and the optical aberration of L_2_ was high when Φ_4_ took values near the limit position.

**Figure 7:**
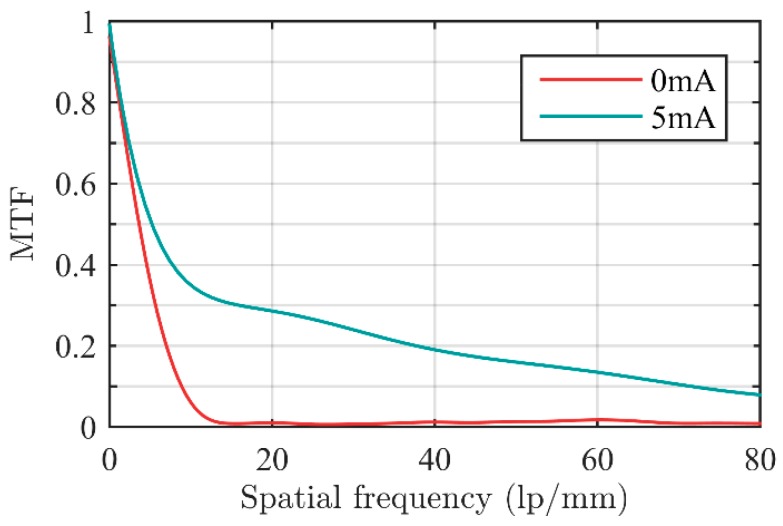
MTF curve of the zoom system when I_2_ took 0 mA and 5 mA, respectively.

In order to achieve high image quality in the whole zoom process, we set I_2_ = 5 mA and adjusted L_1_ to compensate for L_2_. MTF diagram is shown in Figure 7. Parameters of the zoom system when I_2_ takes 5 mA are shown in Table 4.

In the following discussion, we take the state when I_2_ = 5 mA as the initial state.

### 3.3. Electrical Performance Testing

In the process of measuring, L_2_ was adjusted actively to change EFL of the zoom system when L_1_ supplied compensation to get clear images. The relationship between I_1_ and I_2_ can be calculated from Equations (1) and (2).

**Figure 8:**
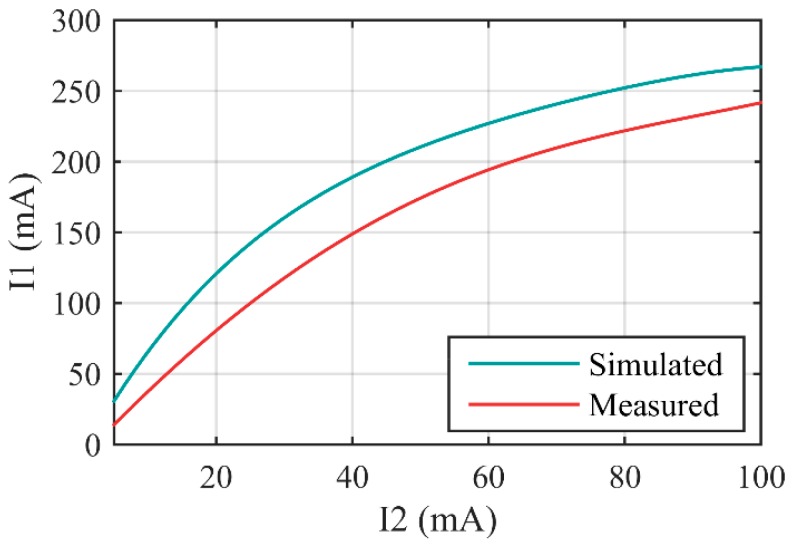
L_1_’s control current (I_1_) as function of L_2_’s control current (I_2_).

We set I_2_ to a value, coarsely adjusted I_1_ till we got clear image on computer, and then tightly adjusted I_1_. Range of tight adjustment was usually less than ±4 mA in actual measurement. We could obtain the real-time MTF of the zoom system with the software written by ourselves on computer. In fact, the MTF curve had no obvious change when we adjusted I_1_ in ±2 mA range. For I_1_, this was an acceptable error range. Then we chose the value of MTF when spatial frequency was 20l p/mm as the image quality factor (Q). We considered the zoom system to achieve highest imaging quality when Q got maximum, and I_1_ would be recorded. The results of simulation and measurement are shown in Figure 8. Although there were differences between simulated curve and measured curve, we could easily obtain I_1_ as function of I_2_ by curve fitting. This enabled the zoom system to realize precise electric control. Both the simulated and the measured curves could be fitted well by three-order polynomials, and the correlation coefficients were larger than 0.999. This meant that we only needed a small amount of addition and multiplication operations to obtain I_1_ by I_2_.

Optical power of the liquid lens was the only variant in the zoom system. Although in actual application on ground vehicle, it needed time to calculate I_1_ and I_2_ for specific EFL, the calculation time was usually at a microsecond level for most of the processors since there were only multiply and add operations in the fitting function. Therefore, the zoom speed depended only on the performance of the liquid lens. The response time of the system was less than 2.5 ms, and the settling time was less than 15 ms due to fluid viscosity.

### 3.4. Zoom Ratio Testing

Relationships between EFL, field of view (FOV), object height (OH), paraxial image height (PIH), and object distance (OD) could be obtained refer to Equations (5) and (6).

(5) $$PIH=EFL\cdot \tan(\frac{1}{2}FOV)$$

(6) $$OH=OD\cdot \tan(\frac{1}{2}FOV)$$

We obtained the magnification (m) of the zoom system by imaging a fixed length (l_o_) target. The magnification could be calculated refer to Equation (7) by counting the pixels (n) when the pixel size (μ) of the CCD camera was known.

(7) $$m=n\cdot \mu /l_{o}=PIH/OH$$

Thus from Equations (5)−(7), we can obtain EFL refer to Equation (8).

(8) EFL=m⋅OD

**Figure 9:**
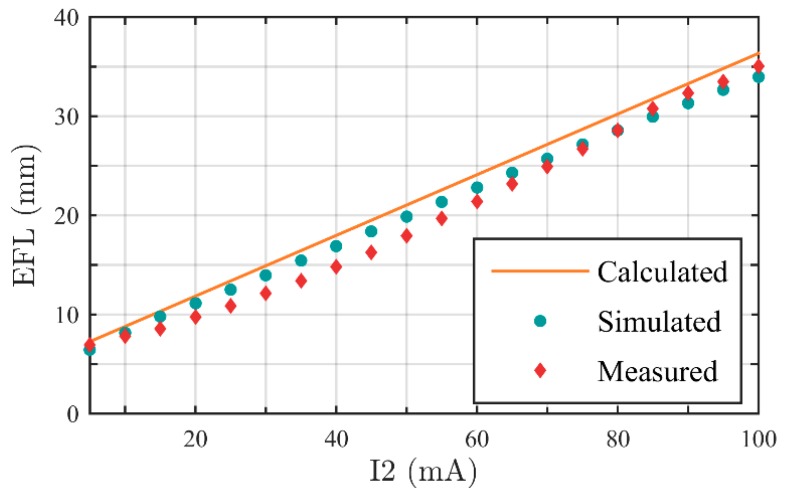
EFL comparison among calculated value, simulated value, and measured value.

The measured and simulated results of EFLs are shown in Table 5, and the EFL comparison diagram of calculated value, simulated value and measured value is shown in Figure 9. An approximate linear relationship exists between EFL and I_2_. Thus, we can easily determine I_2_ to achieve specific EFL in actual application. As presented in Section 3.3, I_1_ could be calculated out by I_2_ through the fitting function. Thus far, issues of determining control parameters to achieve specific EFL are completely solved.

**Table 5:** Comparisons between simulated EFL and measured EFL.

I_2_ (mA)	Simulated EFL (mm)	Measured EFL (mm)	Difference (mm)
5	6.46	6.93	0.47
10	8.14	7.81	−0.33
15	9.78	8.58	−1.20
20	11.12	9.77	−1.35
25	12.52	10.85	−1.67
30	13.97	12.15	−1.82
35	15.43	13.38	−2.05
40	16.91	14.80	−2.11
45	18.39	16.28	−2.11
50	19.88	17.95	−1.93
55	21.35	19.67	−1.68
60	22.82	21.40	−1.42
65	24.28	23.19	−1.09
70	25.72	24.91	−0.81
75	27.15	26.7	−0.45
80	28.56	28.55	0.01
85	29.94	30.77	0.83
90	31.31	32.31	1.00
95	32.65	33.46	0.81
100	33.97	35.06	1.09

Therefore, the measured EFL range of the zoom system is 6.93 mm to 35.06 mm, the zoom ratio is 5.06:1. When I_2_ = 5 mA, EFL takes minimum, at this moment Φ_2_ = 0.0087 mm^−1^ and Φ_4_ = 0.0185 mm^−1^, the FOV is 40°. When I_2_ = 100 mA, EFL takes maximum, Φ_2_ = 0.0192 mm^−1^ and Φ_4_ = −0.0100 mm^−1^ at this moment, the FOV is 8°.

**Figure 10:**
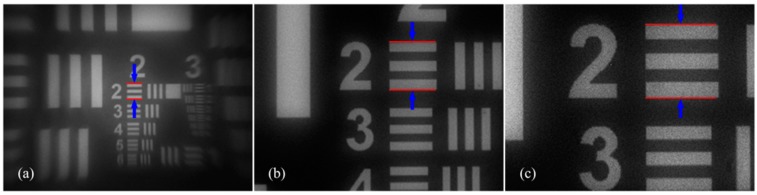
Images captured in different EFL. (**a**) wide end, when I_2_ = 5 mA, 18 pixels between two red lines; (**b**) middle phase, when I_2_ = 60 mA, 59 pixels between two red lines; (**c**) tele end, when I_2_ = 100 mA, 91 pixels between two red lines.

Images captured in different EFL are shown in Figure 10a–c show the wide end, the middle phase, and the tele end respectively. In wide end, EFL is 6.93 mm, the zoom system has the widest FOV, and there are 18 pixels between two red lines shown in Figure 10a. In tele end, EFL is 35.06 mm, the zoom system is most detailed, and the number of pixels between two red lines shown in Figure 10c increases to 91. We can also get the conclusion of a 5.06:1 zoom ratio. A middle phase is presented too, in order to make readers observe the zoom process from wide end to tele end visually.

### 3.5. Zoom Precision Testing

**Figure 11:**
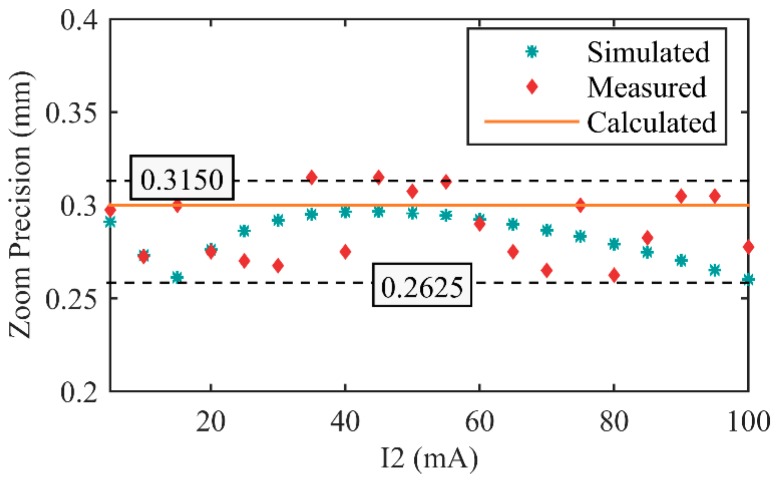
Zoom precision comparison among calculated, simulated, and measured values.

In order to measure the zoom precision of the system, we set I_2_ to a value firstly, measured EFL at this moment. Then we adjusted I_2_ to increase 1 mA, and measured the variation of EFL. In the above, we had simulated the test process. The simulated results had been shown in Table 3. The measured results are shown in Table 6, and diagram of comparison among calculated, simulated, and measured results is shown in Figure 11.

**Table 6:** Measured zoom precision.

I_2_ (mA)	Zoom Precision (mm)	I_2_ (mA)	Zoom Precision (mm)
5	0.2975	55	0.3125
10	0.2725	60	0.2900
15	0.3000	65	0.2750
20	0.2750	70	0.2650
25	0.2700	75	0.3000
30	0.2675	80	0.2625
35	0.3150	85	0.2825
40	0.2750	90	0.3050
45	0.3150	95	0.3050
50	0.3075	100	0.2775
Average	0.2885	RMS	0.0181

Zoom precision of the realized zoom system is approximately uniform in the whole zoom process. The average of measured zoom precision is 0.2885 mm, near to the calculated value 0.3 mm, and all of measured values are in the range of ±0.0265 mm to average zoom precision, RMS of the measured zoom precision is 0.0181 mm. Parameters of the realized lens are consistent with the calculated and simulated values.

Therefore, there are 96 discrete EFL states in the entire zoom range, spaces between adjacent states are approximately uniform to be 0.2885 mm. Because the EFL states are so compact, we can consider the realized system as a continuous zoom system.

### 3.6. Imaging Quality Testing

**Figure 12:**
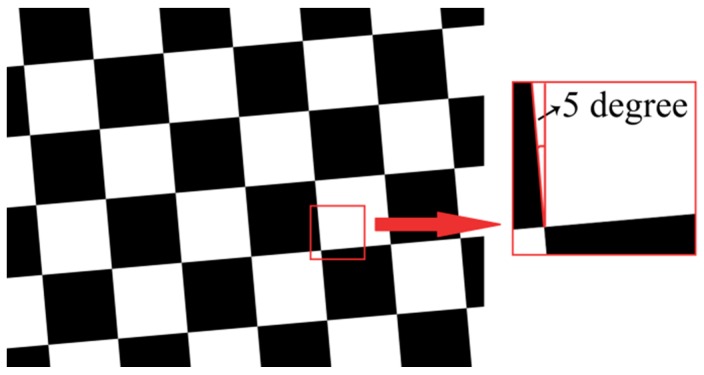
Structure of the target used to test the imaging quality.

We used MTF to evaluate the imaging quality of the zoom system. The test target used in imaging quality testing is shown in Figure 12, similar to a black and white chessboard. The tilt of edge was set to 5° according to ISO12233.

**Figure 13:**
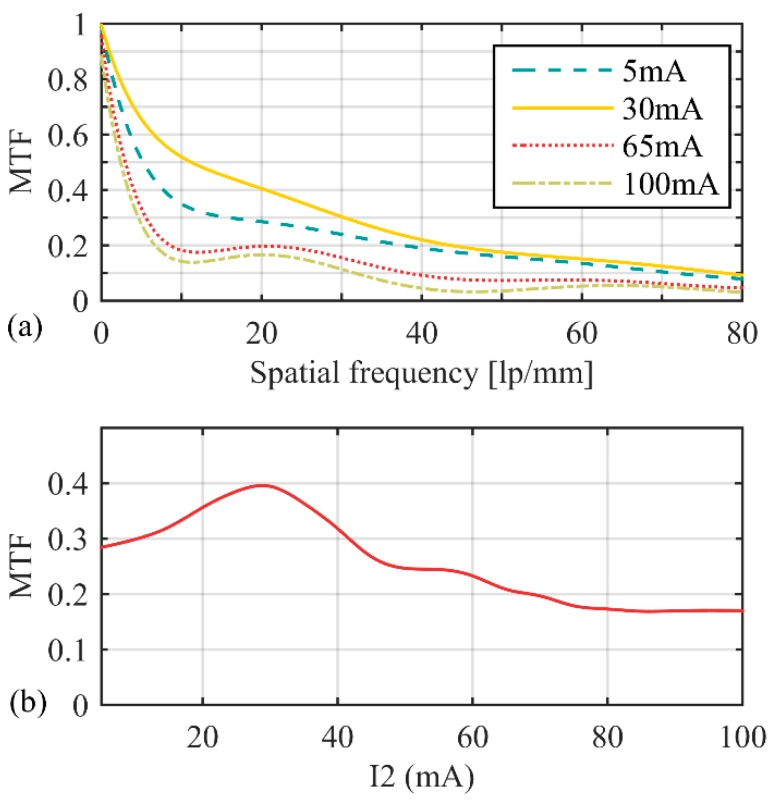
MTF of the zoom system. (**a**) MTFs were measured when I_2_ took different values; (**b**) relationship between MTF and I_2_ at 20 lp/mm.

In the test process, we selected a region-of-interest (ROI) that contains a slanted-edge in the beginning, then obtained the Edge Spread Function (ESF) by the use of knife-edge method and got the Line Spread Function (LSF) through the differential of ESF. The MTF could be computed by a Fast Fourier Transform (FFT) of LSF. MTFs were measured when I_2_ took different values. The results are shown in Figure 13a.

At 20 lp/mm, the relationship between MTF and I_2_ is shown in Figure 13b. The zoom system got the highest imaging quality when I_2_ = 30 mA, MTF reached 0.40 at 20 lp/mm. When I_2_ > 90 mA, MTF reduced to 0.17 at 20 lp/mm.

## 4. Conclusions

In this paper, we designed and realized an electrically tunable four-group stabilized zoom system by using two liquid lenses. The zoom equations were established by using Gaussian bracket method. L_1_ and L_2_ were used as the second and the fourth lens group that were focal length variable. The calculated EFL range was 7.27 mm to 36.34 mm while the zoom precision was calculated to be 0.3 mm. We simulated the zoom system, got the simulated EFL range was 6.46 mm to 33.97 mm and the zoom precision was 0.283 mm. Then we made a series of experiments to verify the performance of the realized system. Response time of the realized zoom system was less than 2.5 ms, and the settling time was less than 15 ms. The measured EFL range was 6.93 mm to 35.06 mm, the zoom ratio was 5.06:1. When Φ_2_ = 0.0087 mm^−1^ and Φ_4_ = 0.0185 mm^−1^, EFL got the minimum value, the FOV was 40°. When Φ_2_ = 0.0192 mm^−1^ and Φ_4_ = −0.01 mm^−1^, EFL got the maximum value, the FOV was 8°. The measured EFL adjust precision was 0.2885 mm, the RMS of precision was 0.0181 mm. MTF was used to evaluate the imaging quality of the zoom system. The range of MTF was 0.17 to 0.40 at 20 lp/mm. The lens realized in this paper had only four groups of elements, and none of them was motorized, thus it had good anti-shake performance. It was applicable to many fields e.g., ground vehicles used in a field-environment, and was also suitable for applications in high speed vehicles and fast moving target tracking as a result of the good electrical performance.

However, the gravity influence of liquid lens was not considered in this paper. Although in the experiments, no apparent gravity effect had been observed, it would exist in theory. In fact, gravity effect in the stationary state had been contained in the parameter “wavefront error” in the datasheet of the liquid lens, the wavefront error was less than 0.5λ. In practice, we need to consider the system working in doubling even tripling gravitational acceleration environments. We will do more research on this issue in future works.
